# Public values to guide childhood vaccination mandates: A report on four Australian community juries

**DOI:** 10.1111/hex.13936

**Published:** 2023-12-28

**Authors:** Chris Degeling, Julie Leask, Katie Attwell, Nicholas Wood, Annette Braunack‐Mayer, Kerrie Wiley, Paul Ward, Stacy M. Carter

**Affiliations:** ^1^ The Faculty of Arts, Social Sciences and Humanities, Australian Centre for Health Engagement, Evidence and Values University of Wollongong Wollongong New South Wales Australia; ^2^ Faculty of Medicine and Health, School of Public Health The University of Sydney Sydney New South Wales Australia; ^3^ School of Social Sciences The University of Western Australia Perth Western Australia Australia; ^4^ National Centre for Immunisation Research and Surveillance Westmead New South Wales Australia; ^5^ Research Centre for Public Health, Equity and Human Flourishing Torrens University Australia Adelaide South Australia Australia

**Keywords:** immunization policy, mandatory vaccination, public health ethics, public participation, social values

## Abstract

**Objective:**

Governments use vaccination mandates, of different degrees of coerciveness, to encourage or require childhood vaccination. We elicited the views of well‐informed community members on the public acceptability of using childhood vaccination mandates in Australia.

**Methods:**

Four community juries were conducted in Canberra, Launceston, Cairns and Melbourne, Australia between 2021 and 2022. We recruited 51 participants from diverse backgrounds, genders and ages through random digit dialling and social media. Two juries were held in metropolitan areas, and two in regional/rural settings. Outcome measures included jury verdicts and reasons in response to structured questions.

**Results:**

All juries were concerned about collective protection and individual rights but prioritised the former over the latter. A majority in all juries supported mandates but juries disagreed with respect to the appropriate mandate types. All juries endorsed using the least restrictive or coercive means to encourage vaccination (providing incentives or education, e.g.) before imposing penalties such as financial losses and school exclusions. The overriding view was that it is fairer to place a direct burden on parents rather than children and that mandates should be designed to avoid inequitable impacts on less advantaged groups in society. Many jurors found conscientious objection acceptable as a controlled option for resolute refusers, provided that overall vaccination coverage remains high.

**Conclusion:**

This paper gives policymakers access to the reasons that Australians have for supporting or opposing different mandates under conditions of high knowledge, understanding and deliberation regarding policy options. Sustaining high rates of vaccination requires high levels of co‐operation between governments, public health actors and the public. Our findings highlight the importance of considering public values in the design and implementation of vaccination mandates.

**Patient and Public Involvement:**

We sought input from individuals who did and did not vaccinate during the study design. The views and perspectives of nonvaccinating parents were presented in the evidence to juries. We deliberately excluded nonvaccinating individuals from participating, as the divisive and often hostile nature of the topic, and their minority status, made it difficult to ensure they would feel safe as members of the jury without overrepresenting their perspective in the sample. Two related projects engaged directly with these parents.

## 1 INTRODUCTION

Vaccination benefits individuals and communities by providing protection against disease and reducing disease transmission. Therefore, a high level of vaccination is usually conceived as a common good, in that it meets the shared interests of the community. A shared set of social norms and moral obligations around childhood vaccination can serve the community by establishing and maintaining collective protection against vaccine preventable disease (VPD). Despite the success of vaccination as a public health measure, levels of coverage can stagnate or decline. Among the suite of measures aiming to increase vaccination coverage,^1^ mandatory vaccination policies (mandates) can be effective in certain contexts.^2^ Mandates impose requirements regarding vaccination and enact a consequence or penalty for those who do not comply.^3^ Mandating vaccination, when it works, can increase civic fairness by limiting free‐riding on the immunity of others, promote distributive justice by widening the reach of vaccine protection and safeguard those most likely to be harmed by vaccine‐preventable disease.^4^, ^5^

However, mandates also have negative social and ethical dimensions, and can be problematic. Mandates can undermine liberty, autonomy, parental discretion, family privacy and public trust.^6^ The coercion associated with mandates can undermine valid consent. Vaccine mandates can cause reactance—anger at being required to take a vaccine.^7^, ^8^ When introduced amidst poor service access and system failures, they may not produce their intended effects.^9^ Relatedly, mandates can intensify disadvantage, since those unvaccinated due to lack of access (rather than choice) are also penalised.^10^ Where the mandate's consequence for nonvaccination is removal of access to education, they deny children equal access to education, limit parents' workforce participation (disproportionally affecting women) and reduce access of at‐risk children to an enriching social and educational environment.^3^, ^11^

Vaccine mandates are designed to minimise vaccine refusal. However, vaccine refusal is complex, and mandates may not have the intended effect in vaccine‐refusers. In high‐income settings, around 2%–3% of parents reject routine childhood vaccines.^12^, ^13^ Their reasons can include previous negative experience with vaccines or medical care, concerns about vaccine safety, doubt about the effectiveness or necessity of vaccines and preference for alternative health approaches^14^; Australian research has reported wide variation in vaccine‐rejection journeys, where parents’ views and actions can be driven by a desire to protect their child and grounded in deeply held health and parenting beliefs.^8^, ^15^

In Australia, childhood vaccination is publicly funded. The population‐level coverage figures are high and stable, with over 94% of 5‐year‐old children fully vaccinated.^16^ It is not known what proportion of the remaining 5% come from vaccine‐refusing families. Vaccine coverage is as low as 78% in some geographic areas,^17^ increasing risk of VPD outbreaks in these localities. The Australian federal government has set a vaccine schedule by age.^18^ Since 1998, the federal government has provided family benefit payments and childcare subsidies only for children who are up to date with the schedule.^19^ At first, parents who lodged a formal ‘Conscientious Objection’ to vaccination could still access these entitlements; this exemption ended in 2016. Australia now has some of the strictest vaccination mandates in the world, operating at the federal and state level. Since 2014, four Australian states (New South Wales; Victoria; Western Australia and South Australia) have tightened the access of undervaccinated children to childcare and preschool services via ‘No Jab, No Play’ policies (Table S1). These policies are popular with the electorate and parents, with no significant public opposition.^20^

Decisions about what measures governing authorities should take to increase vaccination rates in a population are not just a matter for experts.^21^ The perceived legitimacy and public acceptability of measures to encourage parents to vaccinate their children depend on the extent to which they align with community values.^22^ This can be facilitated by structured dialogue between members of the public, relevant experts and decision‐makers to develop ethically and legally defensible justification for vaccine programme design and operation. We report on four community juries convened to consider the acceptability and legitimacy of using mandates to enhance childhood vaccination rates in Australia. Community juries have been used in Australia and elsewhere to consider issues surrounding vaccination and infectious disease control and prevention.^23^, ^24^, ^25^, ^26^ Our aim was to ascertain the conditions under which well‐informed community members would or would not accept different types of measures to encourage parents to vaccinate their children, and why.

## 2 METHODS

Deliberative methods arise from political theory, enact an ideal form of democracy, and are intended to have direct and indirect consequences in governance.^27^ In a community jury, 10–15 members of the public meet for 2–5 days to carefully examine an issue of public significance.^28^ The deliberative process is designed to extend the thinking of participants beyond their own interests to incorporate whole‐of‐population good and the collective needs of the community.^27^ While a small group cannot represent the population, participants are recruited to capture a diversity of experiences and backgrounds,^29^ and so can suggest what an inclusive and informed public would advise, given factual information and time to deliberate.^28^

Community jury methodologies assume people can think rationally and change their views should the evidence warrant it.^30^ Community juries are usually directed to consider a specific issue—typically formulated as a ‘charge’ (as in a court hearing). Jurors hear from a variety of expert witnesses, question those witnesses, and deliberate together on the issue.^28^ To be considered robust and reliable deliberative processes must (at a minimum)^30^, ^31^:

1. provide participants with balanced, factual information;
2. ensure a sufficiently diverse range of potentially conflicting, minority, and marginal perspectives are considered and
3. create opportunities for free and open discussion and debate within and between citizens, experts and/or policy actors, to challenge and test competing claims.

Consensus is encouraged but not required. Juries thus reflect the likely acceptability and public legitimacy of different policy alternatives, and provide an indication of what an informed public think should be done to address a specific issue.^28^

### 2.1 Study settings and contextual information

We convened four community juries: Jury 1 in Canberra (ACT), Jury 2 in Launceston (Tasmania), Jury 3 in Cairns (Queensland) and Jury 4 in Melbourne (Victoria)—representing regional and metropolitan populations.^32^ The juries were held in four state or territory jurisdictions within which governments have taken different policy approaches regarding mandates—see Table S1.^33^ All were held over 2 days (Saturday to Sunday). Because of public health orders and other impacts from the COVID‐19 pandemic, the juries were conducted over 13 months between May 2021 and June 2022. We asked the juries to address a set of questions relating to the following charges (see Figure S1 for full details):

Do you think mandates (a requirement to vaccinate with a consequence for not doing so) should be applied to parents who do not vaccinate their children according to the Australian schedule?

The jury charge changed between CJ#1 in 2021, and CJ#2–4 in 2022 to incorporate a question about COVID vaccination, reflecting jurors' direct experience of pandemic conditions. To assist in interpreting the findings, we map local events during the pandemic in Figure S2.

### 2.2 Juror recruitment

An independent research service was contracted to recruit jury participants, using randomly generated list‐based samples of mobile telephone numbers in specific geographic areas, and a social media advertising strategy targeting those same areas. To reduce selection bias, the social media advertisement was blinded as to the research topic area to avoid overrecruiting participants who had outlier views on childhood vaccination.^34^ Potential jurors were excluded by the research service provider if they were healthcare workers or identified as being opposed to vaccination. The decision to exclude vaccine opponents was based on concerns about their well‐being and safety given the high levels of divisiveness and public hostility that continue to surround this issue in Australia.^35^, ^36^ The larger project these community juries are part of has conducted extensive research with Australian nonvaccinating parents,^7^, ^8^, ^37^, ^38^ and evidence on their experiences and perspectives regarding vaccination and mandates were presented to each jury as evidence. Jurors were selected purposively from the resulting pool of potential participants to promote an approximate 50:50 gender split, to include representation of people with and without children and to ensure a range of ages and socioeconomic and cultural diversity within each jury.^39^ Despite our exclusions, one Cairns juror was highly sceptical about vaccine safety. Jurors were compensated $200 AUD for each day.

### 2.3 Jury procedures

Each jury commenced with an orientation session to introduce the process, the questions for consideration and to seek consent, after participants had read the participant information sheet and had the opportunity to ask questions. A summary of the Jury schedule is provided in Table S2. Day 1 focused on understanding: the individual and population impacts of childhood VPD and COVID‐19; the epidemiology of and policy responses to VPD and COVID‐19 in children in Australia; VPD and COVID‐19 vaccinology including safety surveillance and licensing; vaccine acceptance, refusal, and uptake; the design and implementation of vaccine mandates and ethical perspectives on different responses to parental vaccine refusal. For the last hour of Day 1 and first hour of Day 2, jurors reflected on, discussed and debated the evidence, aided by a facilitator. The facilitation team did not express a position on mandates at any time during the process. Facilitation was positioned neutrally, and focused on promoting constructive dialogue and fair interaction amongst jurors. Juries then deliberated for 90 min, without facilitation, to reach a verdict on the questions posed. The verdicts, underpinning reasoning and dissenting views were then reported to the research team in a final facilitated feedback session. Our research and reporting processes were cross‐checked against the CJCheck protocol.^40^ Further detailed information on jury procedures and the evidence provided by experts can be found in Table S3.

### 2.4 Data collection

The four deliberative groups (juries) comprise the units of analysis. All jury deliberations (facilitated and unfacilitated—see Table S2) and expert question and answer sessions were audio‐recorded and transcribed. To track changes in the positions held by individual jurors, participants completed an anonymous ballot at four time‐points during jury proceedings; each respondent's four surveys were anonymously linked. Jurors also completed a process evaluation exit survey at the conclusion of each event. During the final session on Day 2, a researcher/facilitator recorded the verdict as a tally, and the reasons jurors had for their final votes on a flipchart. Each point was reviewed by the jury to ensure accuracy.

### 2.5 Data analysis

Subsequent to each jury, the transcripts of audio‐recordings were coded using inductive qualitative analysis techniques by Author 1 and a research assistant.^41^ The purpose was to enhance our understanding of the juror's reasons that underpinned the final verdict, by identifying, cross‐comparing and tracking the mobilisation and effect of key concepts and arguments put forward during the deliberations. Differences in interpretation by coders were resolved by consensus. In what follows, the characteristics of each jury are described; a summary of jurors' own descriptions of the reasoning that underpinned their responses to the questions is then provided.

## 3 RESULTS

We report on the verdicts of the four community juries and the reasons given for their decisions. We consider the potential effects of pandemic policies on the different juries in the subsequent discussion.

### 3.1 Participant characteristics

Fifty‐one jurors were recruited across the four study settings (Table S4). All juries included participants who identified as male or female and a range of ages, but those in Launceston and Cairns had more female participants. Each jury had a mixture of participants with and without children—about two thirds were either currently caring for, or had previously cared for, children. Jurors in all study settings had a range of levels of educational attainment, except Launceston, where participants were more educated than elsewhere. Participants in each jury resided in postcodes representing a range of ‘socioeconomic indices for areas’ scores (a measure of average socioeconomic status [SES] of local areas) largely consistent with those of each jury setting.

### 3.2 Community jury verdicts

#### 3.2.1 Part A: Mandating childhood vaccinations

All four juries voted in support of governments applying mandates to parents who do not vaccinate their children (Table 1): in Canberra (CJ#1‐2021) with a slim 7 to 6 majority; in Cairns (CJ#3‐2022) by 13 to 1 and in Launceston (CJ#2‐2022) and Melbourne (CJ#4‐2022) by consensus verdicts. The results of the time‐point ballots (Table S5) demonstrate that the balance of the vote changed throughout each event, with support for mandates softening significantly in Canberra after deliberation but strengthening at the same point in proceedings in the other settings.

**Table 1:** Final vote of the four community juries.

	CJ1 Canberra (*n* = 14^a^)		CJ2 Launceston (*n* = 10^a^)		CJ3 Cairns (*n* = 14)		CJ4 Melbourne (*n* = 13)	
	For	Against	For	Against	For	Against	For	Against
Part A								
Mandates should be applied to parents who do not vaccinate their children according to the Australian schedule	7	6	9	0	13	1	13	0
If mandates are enacted ‐ which penalties should be applied when parents do not vaccinate their children according to the Australian schedule?								
Not being allowed to access family assistance payments	2	11	9	0	6	8	11	2
Not being allowed to enrol children in early years education	5	8	9	0	7	7	6	7
Not being allowed to enrol children in primary or secondary school	2	11	0	9	4^b^	9^b^	7	6
If mandates are applied, should a conscientious objection exemption (the right to refuse based on moral or religious convictions) be available?	13	0	9	0	6	8	3	10
Part B								
If mandates are applied, should they also encompass COVID vaccination for children?	Not applicable		0	9	6	8	7	6

^a^ One participant unable to attend the second day because of illness.

^b^ One participant abstained from this vote.

##### Jurors' reasons for their responses to Part A

Almost all jurors supported maintaining high rates of vaccination to stop VPDs. Jurors who supported using mandates to achieve this goal gave similar reasons across the study settings, although different mechanisms were preferred in different locations. Broadly, mandates were supported because: (1) based on the evidence presented, they were expected to protect the interests of the community by promoting disease prevention and elimination; and (2) promoting collective action to achieve this common good was important. Mandates, therefore, served the purpose of driving greater compliance with childhood vaccination schedules to sustain and protect a fundamental building block of a fair and healthy society.

Support for mandates in all the study settings was conditional. Jurors said mandates should never be stand‐alone measures but should form part of a suite of co‐ordinated interventions to support parents to make good decisions, access vaccination services or both. Including mandates in this suite was justified because small differences in vaccination rates can have major impacts, especially for children with greater vulnerability to infectious diseases. Jurors emphasised that their goal was not to punish vaccine‐refusing parents but to encourage people to take responsibility for one another and vaccinate for the benefit of the community. A strong emphasis was placed on educating parents about the importance of immunisation. This engagement must be community based and flexible. Other strategies should be pursued, such as increasing support and education for parents and raising community awareness by making differences between local vaccination rates public. All the findings reported below should be interpreted in the context of this background recommendation.

A minority of jurors in Canberra and Cairns voted against mandates, believing that mandates could be counterproductive and make some people less likely to vaccinate. In Cairns, in particular, where one juror expressed significant doubts about vaccine safety and described themselves as prochoice, jurors were concerned mandates could split and segregate people, promoting discrimination. More broadly, because Australian mandates target family income (No Jab No Pay) and access to care and education (No Jab No Play), jurors in Canberra, Cairns and Melbourne also expressed concerns that mandates affect children, not just their parents, with disproportionate impacts on families of lower SES. Therefore, for these jurors, it was more just and more persuasive to incentivise vaccination rather than penalise rejection.

When asked to indicate which measures should be applied to parents who refuse vaccination, all four juries came to different positions, with none of the measures strongly supported across all four groups (Table 1). Their reasons for favouring or opposing particular mandate policies are described below.

##### Financial penalties (No Jab No Pay)

A clear majority of jurors in Launceston and Melbourne supported removing access to family assistance payments for vaccine refusers. By contrast, this measure was only supported by a minority of jurors in Canberra and Cairns.

Jurors in favour argued that the loss of income was a powerful motivator to encourage parents who are late in getting their children vaccinated. This penalty would be triggered early in a child's life, prompting vaccination when they are most vulnerable to VPDs. Many jurors argued that imposing financial penalties on parents was the least‐worst option for the child. Specifically, reducing the parent's income was better than restricting the child's education.

Jurors opposed to financial penalties on parents—the majority in Canberra and Cairns—argued this measure was discriminatory and produced unequal impacts. Their key concern was fairness: that parents and children in lower SES families are disproportionately affected by financial penalties.

##### Exclusion from day care/early childhood education (No Jab No Play)

Exclusions from early education was less supported than financial penalties. Only the Launceston jury were in strong support; the other juries were split or narrowly voted against this measure.

Jurors supporting early education exclusions focused on the risks posed by undervaccinated children to their peers. They argued the children of parents doing the right thing should be protected from the choices of nonvaccinating parents. Jurors who supported the measure also argued it was more equitable than financial penalties because it would impact higher SES parents.

Jurors opposing early education exclusion were concerned about impacts on children's social development and learning. This was seen as a significant harm with potential long‐term impacts, and a greater harm than financial penalties. These jurors argued children should not be permanently penalised for their parents' decisions.

##### Exclusion from primary and secondary schooling (compulsory education)

Exclusion from compulsory education, which is not a policy in Australia, was supported by a majority only in the Melbourne jury (see Table 1) and opposed, on balance, in the other three juries.

Jurors who supported compulsory education exclusions argued that letting unvaccinated children into school settings increased the risk of infectious disease outbreaks. Jurors in Cairns and Melbourne, in particular, were also concerned about the disruption outbreaks caused to the education and capacity to work of others.

Jurors who were against this measure argued that for children over 5, education needs to be put first. Allowing children of a nonvaccinating parents to attend school was also seen as a means of exposing them to different opinions and worldviews—such that they might find out about the benefits of vaccination and decide to vaccinate themselves.

##### Should a conscientious objection exemption be available?

The first two juries in Canberra and Launceston came to a consensus that conscientious objection should be permitted; whereas the third and fourth juries voted against permitting conscientious objection, by a narrow majority in Cairns and a much stronger majority in Melbourne.

Jurors who supported conscientious objection argued it was important to allow people who were completely opposed to making choices that reflected their core beliefs.

In Cairns and Melbourne—where there was not a consensus, and the issue was discussed at greater length—the minority who supported conscientious objection argued that the high vaccination rates in Australia meant that a small number of objectors did not significantly undermine the greater good. In all juries, the process of parents lodging their objection was seen as creating a point of contact with health authorities and thereby a space for a conversation. The data collected through the process of nonvaccinating parents lodging a conscientious objection was seen as having significant value to public health authorities, helping them to design programmes and support public health actions during VPD outbreaks.

Finally, support for conscientious objections in all settings was contingent on it being very difficult for parents to obtain. Most, if not all, jurors wanted the process of obtaining a conscientious objection to be so onerous it would pose a significant barrier and discourage all but the most committed vaccine opponents.

The majority of jurors in Cairns and Melbourne who voted against providing for conscientious objection held that the common good of vaccination was too important to become contingent on the choices of individuals. Conscientious objection created a loophole that would be unfair to the vast majority who vaccinate.

#### 3.2.2 Part B: Mandating COVID‐19 vaccines for children

The community juries in Launceston, Cairns and Melbourne also deliberated on the question of whether childhood vaccine mandates should include COVID‐19 vaccinations. We asked jurors to consider, based on the available evidence of COVID vaccination effectiveness in children, whether school closures, social distancing and the economic toll on families as direct burdens on children that warrant their inclusion in vaccination programmes. Once again, the juries came to different positions. The Launceston jury voted unanimously against; jurors in Cairns and Melbourne were divided.

Those jurors in Cairns and Melbourne who were in favour of COVID‐19 vaccine mandates for children argued it was important to make sure that elderly populations and more vulnerable groups were as protected as possible. They reasoned COVID‐19 vaccines offered some protection to the child from severe illness and could potentially reduce socioeconomic disruptions caused by large outbreaks. The jury in Melbourne, who had been locked down for significant periods in 2020 and 2021, also took a position that severe acute respiratory syndrome coronavirus 2 was highly unpredictable. Even though current variants and vaccines were not as impactful for children, the next variant could be much worse. Jurors reasoned that vaccinations would provide some protection, which in these circumstances was better than none.

Contra this precautionary position, jurors opposed to mandating COVID‐19 vaccination in children focused on children's lower vulnerability to severe disease and low vaccine effectiveness in younger age groups. For this group, there was not enough evidence of benefit to justify coercive measures, and mandates risked undermining public trust.

## 4 DISCUSSION

The results of these four Australian community juries demonstrate the benefits of equipping the lay public with the expertise, skill, and process for considering the merits and costs of coercive public policy. These juries were conducted during the COVID‐19 pandemic and vaccine rollout but focused on the legitimacy of mandating vaccines for *children*. Although there was significant variation in the decisions reached, all four juries supported a core set of commitments.

1. The government should take action to promote and support vaccine uptake.
2. All juries supported the use of some kind of mandate: the disagreement between juries was with respect to what kind of mandate.
3. Broad support for mandates was *conditional*. Mandates should never stand alone: they should be part of a suite of co‐ordinated interventions and incentives. This suite should be flexible, tailored, community‐based and educative.
4. Mandates should be a last resort, applied only after parents have multiple engagements with supportive services.

Vaccine refusal remains highly controversial in Australia.^36^ Recent surveys indicate the vast majority of Australians are strongly supportive of childhood vaccination and the imposition of mandates to maintain high vaccination coverage.^20^, ^42^ The current juries were recruited from this large majority in the population. Our results suggest that, even when informed of potential risks, harms, costs, and benefits, members of the public will, on balance, support childhood vaccine mandates with clear caveats. The variation in jury responses is consistent with what is known: mandates involve challenging ethical and social trade‐offs, and there is often persistent disagreement among experts and publics on which type of mandate should be applied.^11^, ^23^

**Figure 1:**
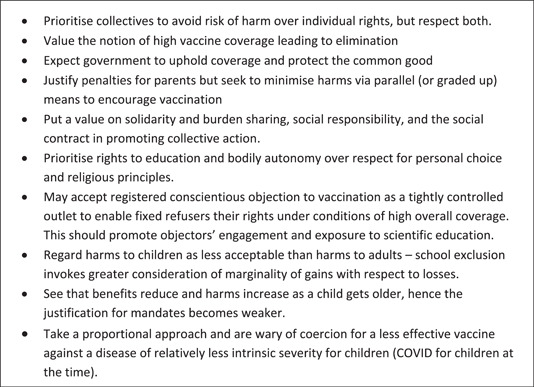
Key considerations—when considering the justification for vaccine mandates, informed Australian publics.

Recent analyses indicates Australian policymakers focus on how mandates can drive up vaccine coverage rates rather than the reasons why they are using them and the various benefits that high vaccine coverage can deliver.^11^ However, in designing and implementing ethical policies to maintain common goods like collective health protection, it is important to consider what communities value. This will require policymakers to shift from outcomes‐focused reasoning to values‐based reasoning, and to articulate their policies using this language. Figure 1 provides a summary of the key considerations of the informed publics (who are generally in favour of vaccination) in this study on this issue. It provides policymakers access to the reasons Australians have for supporting or opposing different forms of mandates. Further ways of taking public values seriously could include examining the effectiveness of a particular mandate in increasing vaccination rates and examining its design, to see whether the same goal can be achieved in a way that addresses the concerns of informed members of the public expressed here.

In deliberations, juries were concerned about both collective protection and individual rights. They prioritised communities over individuals, however, valuing vaccination because it offered collective protection to all children. These juries were convinced that there were good reasons for governments to draw people into mutually beneficial cooperation around childhood vaccination. However, our previous research with nonvaccinating Australian parents found that many reject, or at least are not convinced by, arguments that high levels of vaccination contribute to a common good. These parents reject or doubt the existence of herd immunity, thus believe unvaccinated children are not a threat to others and vaccinating does not lead to collective protection.^7^, ^38^ The common good, in their view, was served by working hard to promote health and well‐being for their children so they did not become sick; and isolating from others when they were sick. Nonvaccinating parents in our earlier research also frequently mobilised the idea of individual rights.^38^ We saw rights‐based arguments in the juries as well, particularly with respect to respect for bodily autonomy and the rights of children to an education, suggesting that Australians may be sympathetic to nonvaccinating parents in this regard. However, nonvaccinating parents in our earlier research also emphasised a right to respect for personal health choices; much less significance was given by jurors to these positions.

There has been one similar public deliberation in Ontario, Canada, with similar findings.^23^ This process asked the jury to define ‘mandatory’, leading to a definition that focused on exclusion from school and other group activities. All 25 jurors agreed that vaccination should be ‘mandatory with medical exemptions’. Five supported and 16 opposed conscientious exemptions, with 4 abstentions. There was a strong emphasis on high‐quality communication with parents, and a majority (18/25) supported graded consequences for parents who did not vaccinate. In contrast to the Ontario process, which allowed jurors to define ‘mandate’, we took a policy‐driven approach, setting out mandate options and asking jurors to respond. This allowed us to provide more detailed reasoning regarding *which* mandates are considered justified. Like the Ontario process, we found that jurors had strong reasons to both oppose and support most measures considered, and were often divided. However because of our method, we were able to show that: (1) support for particular mandates is reliably lower than in principle support for mandating vaccination; (2) limited unvaccinated childrens' access to primary/secondary school is not well‐supported by informed Australians (3) informed australians are divided on whether unvaccinated children should lose access to financial support and preschool education.

Finally, there are also differences in context that need to be considered in interpreting the outcomes of the four community juries: in particular, the effect of direct experience of pandemic conditions and pandemic vaccination programmes. The Canberra jury was held in 2021, before the significant disruptions to society associated with the Delta wave of the pandemic and before COVID vaccines were widely available. The subsequent three juries were held in 2022, when more than 80% of Australians aged over 12 had received two doses of a COVID vaccine and people were adjusting to more relaxed public health settings. The jurors in Canberra, Launceston and Cairns had limited experience of stay‐at‐home orders and home schooling due to COVID‐19 compared to the group in Melbourne, where participants had experienced one of the longest lockdowns globally (Figure S2). Differences between groups may thus, in part, reflect differences in shared lived experience of vaccination programmes. In Melbourne, for example, there was a strong emphasis on harm to others caused by nonvaccination. Canberra jurors, who had not yet experienced significant pandemic community transmission, became less supportive of mandates after deliberation, while in study settings where pandemic impacts were greater, support for mandates increased at the same point in the process. While we can speculate that there are also likely to be general cultural differences between locations which may have influenced outcomes, these are more difficult to evidence reliably.

### 4.1 Study strengths and limitations

When presenting arguments for and against childhood vaccination mandates, one limitation of this study is the asymmetry between the evidence base on the harms to children from preventable infectious diseases on one hand, and the more subtle financial, educational and social harms and burdens that mandates place on families and children on the other. Another limitation is that participants were asked to select between a predetermined range of strategies, while it is possible there are other strategies participants would prefer. However, our design prioritised the direct policy relevance of the questions put to the jurors: that is, we put to the jurors the questions policymakers were asking and wanted answers to. Our recruitment processes were designed to exclude people who refused vaccination. This was to avert potential conflict in the groups but may have rendered jurors less exposed to the views of nonvaccinators.

## 5 CONCLUSION

When considering the acceptability and perceived legitimacy of childhood vaccine mandates, informed publics (who are generally in favour of vaccination) want the instrumental impacts that they believe a mandate will achieve (see Textbox 2). Infringing on rights and causing proportionate harms to nonvaccinating parents and their children is acceptable because of the overriding importance of maintaining high rates of childhood vaccination. The goal of the mandate is to motivate and persuade rather than punish nonvaccinating parents. Key to this is the view that mandates are necessary to drive collective action. But it was morally better to persuade or incentivize parents to vaccinate their children if possible, and people care about the fairness of the consequences of the mandate across a population.

Given that community jury processes on childhood vaccination mandates in Australia and Canada^23^ have produced similar kinds of consistencies and disagreements, we tentatively conclude that the social norm and importance of vaccination is now culturally conditioned in both nations, and perhaps others too. In this context of widespread approval of vaccination, it is hard to obtain agreement on specific measures to promote its uptake when these impose various costs and inequities on those who refuse. When policymakers consider whether to introduce mandates or not, and how to approach questions of flexibility therein, these findings show that informed publics expect considerations such as the balance between beneficial and harmful consequences (for children, parents and the community) that make a policy proportionate, and mutuality and fairness (understood as equality and equity) as being crucial in such decisions.

## AUTHOR CONTRIBUTIONS

Chris Degeling designed the study, ran data collection and analysis processes, and led the drafting and revision of the manuscript. Stacy M. Carter and Julie Leask contributed to study design, ran data collection, participated in data analyses and made significant contributions to the drafting and revision of the manuscript. Katie Attwell, Nicholas Wood, Julie Leask, Kerrie Wiley and Annette Braunack‐Mayer developed the evidence presented to jurors. Katie Attwell, Nicholas Wood and Annette Braunack‐Mayer also participated in data collection and contributed to and revised the drafted manuscript. Paul Ward contributed to study design and revision of the manuscript.

## CONFLICT OF INTEREST STATEMENT

The authors declare no conflict of interest.

## ETHICS STATEMENT

This research was approved by the University of Wollongong Human Ethics Research Committee [2019/244].

## Supporting information

Supporting information.

## Data Availability

Data cannot be shared publicly because of ethics restrictions.
