# Supplementary material for: Public values to guide childhood vaccination mandates: A report on four Australian community juries
Source: Health Expect. 2023 Dec 28;27(1):e13936. doi: 10.1111/hex.13936 (PMC10753634; doi:10.1111/hex.13936)
Supplement: Supplementary file 1 — Supporting information. [file HEX-27-e13936-s001.docx]

**Supplementary Figure 1**: The questions for the Community Juries

During this jury we will ask you to deliberate and decide on one of the following options

**PART A**

Do you think mandates (a requirement to vaccinate with a consequence for not doing so) should be applied to parents who do not vaccinate their children according to the Australian schedule?

NO YES

If you ticked “YES” to the question above, which of the following penalties should be applied when parents do not vaccinate their children according to the Australian schedule?

|  | 1. Not being allowed to access family assistance payments (family tax benefit and childcare rebate and benefit) |
| --- | --- |
|  | 1. Not being allowed to enrol children in early years education (called different things in different states e.g., day care, prep, kindergarten, preschool) |
|  | 1. Not being allowed to enrol children in primary or secondary school. |

If there are mandates applied to parents who do not vaccinate their children, should a conscientious objection exemption (the right to refuse based on moral or religious convictions) be available?

|  | NO |  | YES |
| --- | --- | --- | --- |

**PART B**

Should the same answers you selected in PART A also apply to COVID vaccination for children?

| NO | O |  | YES |
| --- | --- | --- | --- |
|  |  |  |  |

Supplementary Figure 2: Timeline of the 4 Community Juries

The charge considered by the juries changed before CJ2 because of juror’s experiences of pandemic conditions.

Supplementary Figure 3: The question considered by Community Jury 1 in Canberra in May 2021.

The charge considered by the community juries changed after CJ1 as a response to the increasing impacts of the COVID-19 pandemic in Australia. PART B (see Figure 1) was added to the charge in response to the possibility that COVID-19 vaccinations would become available to children under the age of 5 in the near future.

**The charge**

**During this jury we will ask you to deliberate and decide on one of the following options**

**PART A**

Please TICK any of the following mandates if you think they SHOULD be applied to parents who do not vaccinate their children according to the Australian schedule?

|  | Not being allowed to access family assistance payments (family tax benefit and child care rebate and benefit) |
| --- | --- |
|  | Not being allowed to enrol children in day care, kindergarten or preschool |
|  | Not being allowed to enrol children in primary or secondary school |

Should parents be exempted from these penalties if they provide a contentious objection form signed by a medical practitioner?

|  | YES |  | NO |
| --- | --- | --- | --- |

Please tell us the key reasons why you answered in this way

| Supplementary Table 1: Childhood vaccine mandates applied in research settings | | | | |
| --- | --- | --- | --- | --- |
|  | Canberra  (Australian Capital Territory) | Launceston  (Tasmania) | Cairns  (Queensland) | Melbourne  (Victoria) |
| No Jab No Pay | The Federal ‘No Jab, No Pay’ policy requires families to vaccinate their children in line with the national immunisation schedule to be eligible for certain family  support (Centrelink) payments, including childcare subsidies that range from 20–85% of total childcare costs.  The mandate operates across Australia as tied to income tax and family benefit system operated by the Federal Government | | | |
|  | | | | |
| No Jab No Play | Children do not need to be immunised to attend child care services. However, if an outbreak occurs, unimmunised children may be excluded from child care for a period of time. | Children do not need to be immunised to attend child care services. However, if an outbreak occurs, unimmunised children may be excluded from child care for a period of time. | Early education and child care providers have discretionary authority to refuse enrolment or allow access to service if records of vaccination status are requested and not provided. | Early education and child care providers must not enrol a child unless evidence of age-appropriate immunisation or exemption is provided.  Maintain a register of immunisation status/records of each child enrolled.  Request updated certificates within 2 months of the child reaching prescribed age for scheduled vaccination, or at intervals not exceeding 7 months. |
| Key non-medical exemptions to No Jab No Play | Not relevant, as no supporting legislation. | Not relevant, as no supporting legislation. | Not relevant, owing to the discretionary nature of the provisions. | Non medical exemptions can be granted in circumstances where:   - The child and the child’s parent(s) are evacuated from their place of residence due to an emergency (as defined by law). - The child is in emergency care within the meaning of the Children, Youth and Families Act 2005. - The child is in the care of an adult who is not the child’s parent due to exceptional circumstances, such as illness or incapacity. - The child is Aboriginal or Torres Strait Islander. |
| Sanctions for non-compliance |  |  | N/A as provider’s authority is discretionary | Early education and child care providers face fines of up to $20,000, and suspension of a service’s approval to operate. |

Supplementary Table 2: Jury schedule

|  | **Activities** | **Core steps** |
| --- | --- | --- |
| **Saturday** |  |  |
| **08:30-09:15 AM** | **Welcome Coffee and Tea** | - Informal opportunity for participants and facilitation team to meet each other and build relationships |
| **09:15-09:45 AM** | **Orientation Session** | - Understand purpose, information inputs, build skills, build relationships - Time-point ballot 1 taken |
| **09:45-10:30 AM** | **Talk 1: Pre-recorded presentation** followed by question-and-answer session and open discussion between expert on paediatric vaccinology and vaccine program design and jurors | - Information inputs - Group dialogue |
| **10:30-10:45 AM** | **Morning Tea** | - Build relationships - Group dialogue |
| **10:45-11:45 AM** | **Talk 2: Pre-recorded presentation** followed by question-and-answer session and open discussion between expert on vaccine epidemiology and behavioural science and jurors, videos about four case studies online | - Information inputs - Group dialogue |
| **11:45-12:45 AM** | **Talk 3: Pre-recorded presentation** followed by question-and-answer session and open discussion between expert on the design and implementation of vaccine mandates and jurors | - Information inputs - Group dialogue |
| **12:45-01:30 PM** | **Lunch** | - Build relationships - Group dialogue |
| **01:30-02:30 PM** | **Talk 4: Pre-recorded presentation** followed by question-and-answer session and open discussion between expert on bioethics an public health ethics and jurors | - Information inputs - Group dialogue |
| **02:30-CLOSE** | **Facilitated discussion,** debriefing and what to expect tomorrow | - Group dialogue and deliberation - Clarify juror needs for further information inputs - Time-point ballot 21 taken - Debriefing |
|  | **Activities** | **Core steps** |
| **Sunday** |  |  |
| **08:30-09:00 AM** | **Welcome Coffee and Tea** | - Build relationships |
| **09:00-9:30 AM** | **Reorientation Session** | - Process re-orientation - Information inputs - Time-point ballot 3 taken |
| **9:30-10:30 AM** | **Facilitated Deliberation.** | - Group dialogue and deliberation, group decision making |
| **10:30-12:00 PM** | **Open Deliberation** – Un-facilitated and open discussion between jurors | - Group dialogue and deliberation, group decision making |
| **12:00-12:45** | **Verdict Delivery** | - Jurors report on verdict and supporting reasoning |
| **12:45 – CLOSE** | **Closing Session** | - Time-point ballot 4 taken - Participant Feedback and Exit Survey |

| **Supplementary Table 3: Jury Procedures and the Expertise of the Expert Witnesses**  Testimony from four experts was pre-recorded and shown to jurors as video presentations. Experts were selected on the basis of their institutional roles, experience, and expertise, such that they could provide balanced, factual information supporting different expert perspectives on routine childhood and COVID-19 vaccinations, as well as parental vaccine refusal. Each presentation ran for approximately 25-30 minutes. After each video presentation the expert was available by teleconference call or in person to answer jurors’ questions. These question-and-answer sessions, facilitated by a researcher, allowed jurors to clarify or question the evidence and opinions presented. | | | |
| --- | --- | --- | --- |
| Expertise of Witness | | Expert area | Data provided |
| Talk 1 | Paediatric Vaccinology  Vaccination Program Design | Epidemiology and policy responses to vaccine preventable disease in Australia | - Childhood vaccination overview - Brief history of vaccination in Australia - Current vaccination schedules - Disease prevention - Disease incidence pre- and post-vaccination - Impact of low vaccination - Vaccine safety – surveillance and response - COVID-19 and COVID-19 vaccination in children 5-11 |
| Talk 2 | Health social science | Epidemiology & behavioural science | - Vaccination uptake in Australia - Who is under-vaccinated? - Why are they under-vaccinated? - What increases uptake? - What effect did No Jab No Pay have on non-vaccinators? - Behavioural and social drivers of COVID-19 vaccination in children 5-11 |
| Talk 3 | Political science | The design and implementation of vaccination mandates | - Vaccine mandate uses and implications - Main components of vaccine mandates - Australian vaccine mandates (Commonwealth- and State-based) - Mandate exemptions - COVID-19 vaccine mandates |
| Talk 4 | Bioethics, Public health ethics | Research Ethics & Health technology assessment | - Balancing private and public benefits, risks and harms of childhood vaccination - Ethical issues in promotion and enforcement strategies in vaccination - Common goods, parental choice and vaccination |

| Supplementary Table 4: Characteristics of Jury Participants | | | |  |  |  |
| --- | --- | --- | --- | --- | --- | --- |
|  |  | | |  |  |  |
|  |  | Jury 1 (n=14^#^) | Jury 2 (n=10^#^) | | Jury 3 (n=14) | Jury 4 (n=13) |
| Age (years) |  |  | |  |  |  |
| 18-34 |  | 5 | | 3 | 2 | 5 |
| 35-54 |  | 4 | | 4 | 5 | 6 |
| > 55 |  | 4 | | 3 | 7 | 2 |
| Gender |  |  | |  |  |  |
| Male |  | 6 | | 1 | 5 | 8 |
| Female |  | 7 | | 9 | 9 | 5 |
|  |  |  | |  |  |  |
| Highest Educational Attainment |  |  | |  |  |  |
| High School |  | 4 | | 1 | 5 | 6 |
| Trade / Diploma |  | 1 | | 2 | 1 | 1 |
| Bachelor Degree |  | 7 | | 4 | 5 | 5 |
| Postgraduate Degree |  | 1 | | 3 | 3 | 1 |
|  |  |  | |  |  |  |
| Cultural Background/Ethnicity* |  |  | |  |  |  |
| Oceanian (Includes Indigenous Australians) |  | 2 | | 0 | 2 | 1 |
| Southern/Eastern European |  | 3 | | 2 | 1 | 2 |
| South-East Asian |  | 1 | | 0 | 1 | 2 |
| North-East Asian |  | 1 | | 0 | 0 | 0 |
| Southern/Central Asian |  | 0 | | 1 | 2 | 1 |
| North-West European |  | 5 | | 7 | 6 | 6 |
| Peoples of the Americas |  | 1 | | 0 | 1 | 1 |
| Sub-Saharan African |  | 0 | | 0 | 0 | 1 |
| North African and Middle Eastern |  | 1 | | 0 | 1 | 1 |
|  |  |  | |  |  |  |
| Socio-Economic status of suburb** |  |  | |  |  |  |
| Low |  | 0 | | 3 | 0 | 1 |
| Middle |  | 0 | | 5 | 12 | 4 |
| High |  | 13 | | 2 | 2 | 8 |
|  |  |  | |  |  |  |
| Had Children |  |  | |  |  |  |
| No |  | 5 | | 3 | 4 | 6 |
| Yes |  | 8 | | 7 | 10 | 7 |
|  |  | | |  |  |  |
| # 1 Participant unable to attend the second day because of illness | | | | | | |
| * Based on Australian Standard Classification of Cultural and Ethnic Groups (ASCEG) | | | | | | |
| ** Based on Socio-economic Index for Area (SEIFA) | | | | | | |

| Supplementary Table 5: Votes at different time-points during jury proceedings | | | | | | | | | | | | | | | |
| --- | --- | --- | --- | --- | --- | --- | --- | --- | --- | --- | --- | --- | --- | --- | --- |
| Time point | CJ #1 (Canberra) | | | CJ #2 (Launceston) | | | | CJ #3 (Cairns) | | | | CJ #4 (Melbourne) | | | |
|  | Childhood vaccine mandates  For/Against | Conscientious objection  For/Against | COVID vaccine mandates  For/Against | | Childhood vaccine mandates  For/Against | Conscientious objection  For/Against | COVID vaccine mandates  For/Against | | Childhood vaccine mandates  For/Against | Conscientious objection  For/Against | COVID vaccine mandates  For/Against | | Childhood vaccine mandates  For/Against | Conscientious objection  For/Against | COVID vaccine mandates  For/Against |
| #1 Saturday AM  Before evidence delivered | 11/3 | NA | NA | | 10/0 | 7/3 | 7/3 | | 12/2 | 8/6 | 7/5 | | 12/1 | 4/9 | 10/3 |
| #2 Saturday PM  After evidence delivered | 12/2 | NA | NA | | 8/2 | 7/3 | 7/3 | | 12/2 | 11/3 | 11/3 | | 12/1 | 5/8 | 8/5 |
| #3 Sunday AM  After reflection overnight | 6/7* | 9/4* | NA | | 8/1* | 8/1* | 5/4* | | 12/2 | 8/6 | 6/8 | | 11/2 | 4/9 | 8/5 |
| #4 Sunday PM  After deliberation | 7/6* | 13/0* | NA | | 9/0* | 9/0* | 0/9* | | 13/1 | 6/8 | 6/8 | | 13/0 | 3/10 | 7/6 |
| * Juror failed to attend on second day of proceedings because of personal or family illness | | | | | | | | | | | | | | | |
